# The Book World of Medicine and Science

**Published:** 1904-06-11

**Authors:** 


### THE BOOK WORLD OF MEDICINE AND SCIENCE.

THE MEANING OF A MODERN HOSPITAL. By W. BRUCE CLARKE, M.A., M.B.Oxon., F.R.C.S. (London: Longmans, Green and Co. Pp. 47. Price 1s.)

THE author has produced a very useful pamphlet for the layman interested in hospital construction; and even the expert would do well to study it and so familiarise himself with the requirements which modern hospital surgeons think essential. At the beginning Mr. Bruce Clarke boldly admits that the best results have not always been obtained in hospitals. Beyond all doubt this is true, and he illustrates the truth by telling us that the mortality in the hospital at Scutari was 42 per cent., while that in the wooden huts at Balaclava was only 3 per cent.; and it might further have been stated that in the old days of hospital gangrene following amputations in the field, those soldiers who recovered owed their lives to being kept in a whirlwind of fresh air. The modern hospital is, however, a vast improvement on the old ones and excessive mortalities need no longer be dreaded. By modern we mean those built within the last twenty years or so; and as wooden huts designed for one or two patients are impossible in our crowded cities we must content ourselves with these modern specimens in the hope that full advantage will be taken of the constructive improvements of the time. We agree with Mr. Clarke that 4,000 cubic feet of fresh air per patient per hour may not be sufficient in all cases; but it is useless for architects to provide means of ensuring even half that amount so long as medical officers are unconverted as to the advantages of an abundant supply of cool air. It is not only our climatic conditions that stand in the way of this fresh air. It is the old ideas which still prevail in some quarters; and which not only prevent the admission of an abundant supply of fresh air but insist on artificially warming that which is admitted. It is possible that the hospital architect of the future will approach the design of the old Asclepion wards in which the whole of one side was open to the fresh air. Mr. Clarke points out that floor space is more important than mere cubic space in a modern hospital. In the model ward or hospital unit which he gives us, the length is about 100 feet, the width 26 feet, and the number of beds is 24. Here we get about 110 superficial feet per bed, and certainly this is not excessive, although it might pass in an old hospital, but hardly in a new one, unless the perflation of air were very carefully looked to. Then $8\frac{1}{3}$ feet is a small allowance of wall space per bed. Surely 10 feet ought to be the minimum. Apart from this the model ward is good enough except that the beds at both ends, that is 4 beds out of 24, have windows on one side only; but this may be a mistake of the draughtsman in a small-scale plan. The sanitary annexes are well placed and well cut off from the ward; but it would be much better to omit the centre partition of the bath-room and to have the bath-heads turned to the windows so that the baths could be approached from both sides and one end, instead of being jammed against the wall as they are. Then in the opposite annexe the closet-doors are exactly opposite the door of the annexe, and the nurse would have to run the gauntlet of these doors every time she went to the sinks which adjoin the closets. Of course these are faults of detail rather than of construction. At the opposite end of the ward are the sisters' room, the clinical room, kitchen, nurses' W.C., the cupboards and sinks, the day-room, a ward for two beds and a ward for one bed. There is no linen-room and no patients' clothes-room although these are mentioned in the letterpress. As regards the two-bedded room it has windows on the end and on one side, and the windows of the one-bedded ward are similarly disposed, so that in neither case is there proper cross-ventilation; and we look upon the position of the nurses' closet as faulty. Want of space forbids our noticing the useful remarks upon warming and ventilation, and the construction of operating theatres, nurses' homes, etc. Mr. Clarke concludes his interesting pamphlet by saying, "rely upon it, that if a modern hospital ward is destined only to last fifty years, it will be far more economical to construct it soundly, plainly, and without ornament."

BACTERIOLOGY OF EVERYDAY PRACTICE. Medical Monograph Series. No. 2. By J. ODERY SYMES (London: Baillière, Tindall and Cox. Pages 103. Crown 8vo. Price 2s. 6d. net.)

THIS little book has been designed to meet the needs of those practitioners who, not having been able to acquire an intimate knowledge of pathology, are now anxious to become acquainted with such elementary methods of bacteriological investigation as are necessary to them in their everyday work. The objects of the monograph are well put in the introductory chapter as follows:—"(1) To point out in what cases bacteriological examination might help in clinical diagnosis; (2) to describe methods of securing and identifying microscopic specimens such as the student could himself prepare; (3) to give directions for taking cultures and for preserving tissues to be sent to the laboratory." No attempt is made to describe at any length varieties or the various properties of bacteria; indeed the size of the book prohibits such a course—it is essentially a practical handbook for the beginner who has to rely entirely on himself. The opening chapters deal with the various instruments necessary, the formulæ for the preparation of the ordinary stains, and the methods of making and staining film preparations from pus, blood, urine, etc. In succeeding chapters different diseases are dealt with, and the methods of collecting material and recognising varieties of bacteria explained. If there is a fault to be found with the book, it is the scarcity and poorness of the illustrations. They are in our opinion too few in number, and much too diagrammatic, conveying mostly a very poor idea of the original. We would suggest that a few representations of micro-photographs of the common types of organisms would add greatly to the value of the book. Otherwise it is admirable, and should prove of great practical value.

KELLY'S DIRECTORY OF CHEMISTS AND DRUGGISTS, 1904. (Kelly's Directories, Limited, 182–184 High Holborn London, W.C. Price 20s.)

THE present is the tenth edition of this most useful directory and contains within its pages names of some 56,000 persons connected with the chemical and allied trades in the United Kingdom, this being, it is stated, an increase of about 4,000 names as compared with the last edition. Every care seems to have been taken to verify the names contained in the work, and its admirable arrangement and full and accurate indices should render it of great practical value as a work of reference to all interested in or connected with the chemical trades.

DISEASES OF THE APPENDIX VERMIFORMIS. By F. G. LLOYD. (London: John Bale, Sons and Danielson. Price 2s. net.)

THIS is a reprint of a paper read before the West London Medico-Chirurgical Society. It is a small pamphlet reviewing the subject of appendicitis and its treatment. We cannot assert that there is anything new put forward or anything that cannot be found in the ordinary text-books on the subject. To anyone looking up the subject we cannot recommend this pamphlet as likely to afford him any satisfaction.

